# Contraceptive challenges and the transgender individual

**DOI:** 10.1186/s40695-018-0042-1

**Published:** 2018-07-13

**Authors:** A. Francis, S. Jasani, G. Bachmann

**Affiliations:** Clinical Instructor and Academic Faculty Scholar, Department of Obstetrics, Gynecology and Reproductive Sciences, Rutgers Robert Wood Johnson Medical School, 125 Paterson St, New Brunswick, NJ 08901 USA

## Abstract

In recent years public awareness of healthcare disparities experienced by transgender individuals throughout the world have garnered increasing attention within the media and from health advocates. Despite this increasing awareness, a paucity of research data and clinical protocols of care for clinicians continues to exist, especially in regard to the transgender individual’s family planning needs. Clinicians should be on the forefront of promoting strategies that forge a meaningful and collaborative relationship with the transgender man, including as he transitions through to the menopause and his sexual and reproductive healthcare needs. Unfortunately, despite best efforts to address the health concerns of transgender men in midlife, including their contraceptive needs and pregnancy desires, there is currently a paucity of research. Although hormonal contraceptives are not an option for this group of individuals, especially those on masculinizing hormones, IUD’s, both copper containing and progestin containing, should be considered for those with intact pelvic organs. For this group of transgender men with potential for pregnancy who have either completed their family or choose not to give birth, sterilization can be offered. Regardless of where they identify along the gender spectrum, these midlife individuals with potential reproductive potential should have equitable access to and up to date counseling on their contraceptive options. This commentary addresses the contraceptive challenges of the midlife transgender man. (Note: Pronouns used in this article are he/him for cis and transgender men and she/her for cis and transgender women).

## Background

Approximately 0.3% of individuals within the United States and 0.3–0.5% of the global population identify as transgender, however the exact prevalence of this population remains unknown, and their contraceptive needs shold be addressed more actively [1–4]. Though recent public awareness of transgender individuals and their unique health care needs have been addressed within the media and professional educational materials, there is a paucity of research data and evidence based protocols available that could help guide clinicians on the appropriate care regarding specific health challenges facing this population. In fact, centers that provide transgender sensitive care, such as The Center of Excellence for Transgender Health at University of California, San Francisco (UCSF) and Fenway Health, as well as our Gender Center of New Jersey a joint Rutgers and RWJBarnabas Health partnership, have commenced, with the major focus on the health care needs of this population and, for many centers, the health care needs of the LGBTQ population in general.

One health care challenge is providing optimal management of the contraceptive needs for the transgender man with intact pelvic organs who is sexually active with a partner pairing in which a pregnancy can occur. This challenge pertains to not only the younger transgender male, but also to the midlife transgender man, a patient scenario in which many clinicians may not address the patient’s fertility issues at all. Many clinicians will consider midlife transgender men not at risk for pregnancy due to presumed exhaustion of functional follicles in their ovaries, which is not always the case. That is, although some practitioners may devote time to contraceptive options when counseling younger transgender men, such counseling usually is not given the same priority for these individuals during their midlife years because they are often assumed to be infertile.. Until the individual is menopausal or has had a sterilization procedure, there continues to be a risk of pregnancy if unprotected sexual intercourse that involves deposition of sperm in the vaginal canal is occurring.

The symptomatology, signs and age of the menopausal transition for transgender men who have not had affirmation surgery with removal of ovaries is unique to each individual. Therefore, if the midlife transgender man has both intact an uterus and ovaries, he should be considered as having reproductive potential until there is documentation that he is menopausal. The midlife transgender male’s menstrual cycle may be irregular or suppressed due to the exogenous hormones being utilized for gender affirmation and not from perimenopausal changes. Serum Antimullerian Hormone testing that measures ovarian reserve should be considered in at-risk for unwanted pregnancy transgender individuals as well as for those who are considering what pregnancy options they have. Those at-risk individuals who do not desire pregnancy should be given comprehensive contraceptive counseling tailored to their preferences andneeds. For this commentary a literature search through PubMed and Google Scholar was done using key word that include transgender, birth control, family planning, fertility and contraception, with specific attention to these issues in midlife and older women. Of note, when these key words were searched as they specifically pertain to the midlife trans community and their contraceptive needs, there was a lack of data.

### Overview

As in all populations, health maintenance and concerns also are issues of the utmost importance for transgender individuals. The treatment of chronic medical conditions, sexual problems and mental health concerns, in addition to preventative health counseling, are similar in the transgender population as they are in all other populations. However, in addition to many of the universal health challenges facing patients in general, transgender individuals (both men and women) face unique challenges due to their marginalized status, [5, 6]. As noted by Ryan K. Sallans, an internationally known transgender individual and speaker, there are several important points the clinician should be cognizant of when providing care to the transgender population, which he has learned both from his personal transgender experiences as well as from a provider’s perspective [4]. A common theme for optimal transgender health care is the need for individualization of care and the need for taking into account specific barriers to care, including stigmatization and not feeling safe in most health care settings. As well, addressing increased health risks, such as depression, suicidal thoughts and actions, sexual violence and sexually transmitted infections in the transgender population also are important parts of the medical history. The clinician should not assume that the transgender patient will bring up these issues without direct clinician inquiry it As important, all providers should ask transgender men, including those in their midlife years, if they have intact ovaries and a uterus and if they are at risk for pregnancy. Many times a pelvic examination on the individual is not done or a pelvic ultrasound or other pelvic imaging is not available to confirm pelvic anatomy. Further, it should not be assumed that the transgender man is not having sexual exchange with a partner who is capable of fathering a wanted/unwanted pregnancy. During the medical history, midlife transgender men should be assessed regarding if they are at risk and if they are, be asked if they would like to discuss fertility issues, as becoming pregnant may be desired. If the individual states that there is not a desire to become pregnant, then reversible and/or permanent family planning methods should be discussed.

Education and counseling are key, since some clinicians, as well as patients at midlife, believe that pregnancy is not possible or rare. Although midlife is accompanied by decreased fertility, pregnancy may still naturally occur without assisted reproductive technology (ART) intervention. Therefore, midlife transgender men at risk for pregnancy should be told about all contraceptive options that will not interfere with their hormonal affirmation treatment. In those individuals who decide that they want a contraceptive method and have decided on what is best for them, doing necessary testing and then proceeding with the desired method can be completed at that visit or initiated then.

### Barriers to contraceptive Care for Midlife Transgender men

To adequately counsel the midlife transgender man, clinicians must create a safe environment in the medical setting they are providing this care in. This is a major barrier for many transgender individuals who anticipate before the medical encounter that they may be treated in an unprofessional or hostile way by the clinician and/or the health care team. In addition, the transgender man, often feels uncomfortable and not safe in a waiting area populated only with cis gender women or pregnant women if he is being seen in an Ob/Gyn office setting. Providing times, either before or after usual office hours when there is a limited number of patients in the Ob/Gyn waiting area should always be strived for. As well, transgender men should be addressed by all clinical staff and clinicians by their preferred name, which may not be the name listed on the insurance card or medical record or even driver’s license. Correct pronoun use also is key. Many individuals prefer they/them to he/him or she/her. All office staff must be educated on the patient’s preferred name and pronoun. Referring to a patient by the incorrect name and/or pronoun will cause discomfort and may inhibit the patient from further open discussion.

Add to this the discomfort that often accompanies an exchange on sexual and reproductive health concerns. This discomfort is further exacerbated by the social and political backdrop of being stigmatized in the majority of communities that health care settings exist in. Despite the fact that many countries have made significant advances in LGBTQ+ rights in recent years, discrimination and disparities persist and make it more difficult for the midlife transgender man to identity and discuss his sexual and contraceptive needs in a safe environment [7]. Furthermore, many practitioners may not care for a large number of midlife transgender men and therefore are not prepared to fully take a comprehensive history, including a history of sexual violence that has/is directed at the individual, educate them comprehensively on birth control options and manage their contraceptive needs.

Specifically, when addressing the contraceptive needs of the transgender man in midlife, the clinician may not have the experience and training to adequately counsel the patient on their birth control needs. Facts that have to be known are hormonal affirmation medications being used, whether surgery has been done to excise the uterus and/or ovaries and whether the patient is at risk for unwanted pregnancy. Once these patient facts are known, family planning options can be presented to the patient that are individualized to his needs. As well, continuing education lectures and materials as well as collaborating with colleagues who have expertise in this area are optimal ways of becoming more proficient and comfortable in caring for transgender individuals, not only for family planning management, but for overall medical care.

### Addressing contraceptive and fertility care

When looking at family planning issues in general, it is well known that approximately half of all pregnancies annually in the United States are unintended; many of which contribute or causeadverse effects on maternal, infant and family health in addition to economic and societal burdens [8]. Individuals with reproductive capability for fecundability, regardless of preferred gender, who use contraception consistently and correctly, account for only 5 % of unintended pregnancies [1]. Further, for midlife individuals, the CDC recommends that contraception use continue until menopause or at least until 50 to 55 years of age to reduce unintended pregnancies1.

It is evident that contraception is effective; however it is vital that the appropriate method be selected taking into consideration patient preference and the specific health needs and risks of the individual. This is especially important when dealing with transgender individuals who will need full counseling on what is safe for them and what methods will not interfere with their gender affirmation managment.

For transgender men, there is a paucity of data examining pregnancy risk and contraceptive guidelines creating an added level of complexity and difficulty to appropriately deliver care. Based on the limited data available, unintended pregnancy also is a health risk for them. For example, in one study of 41 transgender men, 24% of these individuals experienced unintended pregnancy after transitioning [9]. As already noted, not all pregnancies occurring in the transgender population are unintended. In another study assessing reproductive desires within the transgender population, 54% of individuals had the desire to have children and 37.5% of both transgender men and women considered the use of fertility preservation to achieve their reproductive goals [10].

From the perspective of the clinician, balancing fertility desires in conjunction with contraceptive counseling is a discussion that should be had with all midlife transgender men. The unknown long term risks of the specific medical and surgical modalities used for transitioning and maintaining desired sexual characteristics has to be taken into account as this information is vital in order to provide appropriate contraceptive counseling, Unfortunately, the answer to many questions regarding hormonal management in the transgender patient and its impact on contraception and fertility is not clearly known and therefore many younger transgender individuals should be counseled on fertility preservation options currently available.

### Contraceptive options for the midlife transgender man

The process of transitioning is not a uniform one for every individual. As such, it is quite possible that individuals who identify themselves as transgender men may not, or perhaps even have chosen not to, avail themselves of the male-enhancing capabilities of testosterone. Transgender men also may or may not be interested in surgical procedures to confirm their male gender identity. Therefore, there is a range of gender affirmation interventions in the transgender male population and each individual’s contraceptive needs will be different. There is qualitative evidence indicating an increased likelihood that transgender men are interested in suppression of their menstrual cycle [11]. Therefore, in the transgender man not on masculinizing hormones, amenorrhea could be partially achieved with a progestin containing IUD if reversible contraception is desired, or with endometrial ablation if a more permanent method is desired. It should be noted that endometrial ablation is not considered a permanent form of birth control.

For those on hormones that enhance male characteristics, female hormones in combination oral contraceptive pills, transdermals, injectables (such as Depo Provera) and vaginal preparations (such as the contraceptive) would not be indicated. The most optimal choices for those who have made the decision to permanently eliminate their reproductive potential would be the offer of a bilateral tubal ligation or a placement of an Essure device. The Essure device placement results in tubal occlusion that approximately six weeks to twelve weeks after the device is placed hysteroscopically. Under the appropriate circumstances this procedure has the benefit of being done in an outpatient setting, and as such, patients can avoid the risks associated with general anesthesia. According to the CDC it has a failure rate of 0.5% making it one of the most effective means of contraception [1]. For those who desire bilateral tubal ligation, a discussion of excision of fallopian tubes should be part of the counseling. Recent research on ovarian cancer, and its likely origins in the fallopian tube, has suggested that rather than tubal ligation, the removal of both fallopian tubes should be done instead as this may reduce the individual’s risk of ovarian cancer. 11 [12].

As for transgender men who are unsure of their long term desires for reproductive potential, long acting reversible contraception (or LARC) containing progestins only are the most effective methods of contraception. These devices include progestin containing intrauterine devices (or IUDs) as well as transdermal implantable devices These removable (and reversible devices) are most appropriate for individuals with reproductive potential who have not made (or do not wish to make) long term or irreversible decisions regarding their future reproductive potential. Once again these LARCs are the effective methods of contraception that usually do not interfere with the hormonal affirmation treatment the individual has been prescribed [1]. Barrier methods also are effective, and they are the only means of protection against HIV and other sexually transmitted infections; however, their effectiveness at preventing pregnancy is secondary to LARC in typical use scenarios. However, the transgender man must have an intact vagina to utilize barrier contraceptives. From the patient perspective, because they are associated with use during each sexual encounter, barrier methods are often not acceptable to transgender men.

## Conclusion

Health challenges such as sexually transmitted infections, preservation of fertility, and sexual violence, in addition to contraception are all prominent issues facing all transgender individuals, including midlife transgender men. For transgender men in midlife who have not had a hysterectomy, contraceptive concerns also are an important part of their annual wellness visit and should be included in these encounters. Additionally, the barriers to care, social stigma and limited data regarding transgender health are all deficiencies of the current health care system. Regardless of where a patient identifies on the gender spectrum, their needs and rights to equitable access, up to date knowledge of the contraceptive options and provision of the management best for them should be a part of their medical care.
